# VISPA2: a scalable pipeline for high-throughput identification and annotation of vector integration sites

**DOI:** 10.1186/s12859-017-1937-9

**Published:** 2017-11-25

**Authors:** Giulio Spinozzi, Andrea Calabria, Stefano Brasca, Stefano Beretta, Ivan Merelli, Luciano Milanesi, Eugenio Montini

**Affiliations:** 1 San Raffaele Telethon Institute for Gene Therapy (SR-Tiget), IRCCS, San Raffaele Scientific Institute, Via Olgettina, 58, 20132 Milan, Italy; 2 Department of Computer Science, University of Milano Bicocca, Viale Sarca, 336, 20126 Milan, Italy; 3 National Research Council, Institute for Biomedical Technologies, Via Fratelli Cervi, 93, 20090 Segrate, Italy

**Keywords:** Open source software, Bioinformatics pipeline, Integration site analysis, Gene therapy, High-throughput sequencing, Next-generation sequencing, Workflow

## Abstract

**Background:**

Bioinformatics tools designed to identify lentiviral or retroviral vector insertion sites in the genome of host cells are used to address the safety and long-term efficacy of hematopoietic stem cell gene therapy applications and to study the clonal dynamics of hematopoietic reconstitution. The increasing number of gene therapy clinical trials combined with the increasing amount of Next Generation Sequencing data, aimed at identifying integration sites, require both highly accurate and efficient computational software able to correctly process “big data” in a reasonable computational time.

**Results:**

Here we present VISPA2 (*Vector Integration Site Parallel Analysis*, version 2), the latest optimized computational pipeline for integration site identification and analysis with the following features: (1) the sequence analysis for the integration site processing is fully compliant with paired-end reads and includes a sequence quality filter before and after the alignment on the target genome; (2) an heuristic algorithm to reduce false positive integration sites at nucleotide level to reduce the impact of Polymerase Chain Reaction or trimming/alignment artifacts; (3) a classification and annotation module for integration sites; (4) a user friendly web interface as researcher front-end to perform integration site analyses without computational skills; (5) the time speedup of all steps through parallelization (Hadoop free).

**Conclusions:**

We tested VISPA2 performances using simulated and real datasets of lentiviral vector integration sites, previously obtained from patients enrolled in a hematopoietic stem cell gene therapy clinical trial and compared the results with other preexisting tools for integration site analysis. On the computational side, VISPA2 showed a > 6-fold speedup and improved precision and recall metrics (1 and 0.97 respectively) compared to previously developed computational pipelines. These performances indicate that VISPA2 is a fast, reliable and user-friendly tool for integration site analysis, which allows gene therapy integration data to be handled in a cost and time effective fashion. Moreover, the web access of VISPA2 (http://openserver.itb.cnr.it/vispa/) ensures accessibility and ease of usage to researches of a complex analytical tool. We released the source code of VISPA2 in a public repository (https://bitbucket.org/andreacalabria/vispa2).

**Electronic supplementary material:**

The online version of this article (doi:10.1186/s12859-017-1937-9) contains supplementary material, which is available to authorized users.

## Background

The molecular analysis of the genomic distribution of viral vector Integration Sites (IS) is a key step in hematopoietic stem cell (HSC) -based gene therapy (GT) applications, supporting the assessment of the safety and the efficacy of the treatment [1–5].

IS are retrieved by specialized PCR (Polymerase Chain Reaction) protocols designed to amplify the genomic portions flanking the vector integrated in the host cell genome which are subjected to Next Generation Sequencing (NGS). Sequence analysis performed with dedicated bioinformatics pipelines allows the precisely mapping of the input reads on the reference genome in order to identify the vector/cellular genomic junction positions. Furthermore, it offers the possibility to identify the genes targeted by vector integrations and to evaluate if specific classes (for example oncogenes) are excessively enriched over time. Moreover, since vectors, such as retroviruses and transposons, integrate semi randomly in the genome of host cells, each vector IS is a genetic mark characteristic of each vector-transduced cell and its progeny. This means that retrieved IS can be used to identify and study the behavior of thousands of vector-marked clones. Finally, since the number of sequencing reads of each IS is proportional to the abundance of the cell clone population harboring that IS, it is possible to estimate the clonal population size and thus detect or exclude sustained clonal expansions, a worrisome preluding sign of genotoxicity. Hence, IS analyses are fundamental for monitoring gene therapy safety by detecting early sings of genotoxicity (even before tumor onset) and the treatment efficacy of the treatment in preclinical testing and in GT patients. For these reasons, in depth molecular studies based on IS are required by regulatory authorities for the evaluation of GT products with an increasing level of detail.

Beyond the GT field, integration studies have also a great importance in virology by allowing the study of the clonal composition of HTLV-1 or HIV-1 infected cells and their expansion in patients [6–9]. Moreover, these studies have been fundamental in retroviral and transposon based insertional mutagenesis screenings aimed to discover novel oncogenes and tumor suppressor genes in mice and human studies [10–13].

Although several tools for IS identification have been developed [14–22], the large amount of data generated by NGS technologies poses novel computational challenges which requires high performance algorithms able to provide scalability for long-term studies such as those required in pharmacovigilance for GT trials and in other applications.

To overcome these issues, we developed VISPA2 with the following new features: (1) processing Illumina paired-end reads generated by PCR methods for IS retrieval that use DNA fragmented by restriction enzymes [23, 24] or sonication [25–27]; filtering low quality reads, before and after the alignment, to reduce false positives; (2) improving the precision of IS identification at nucleotide level with a module based on a heuristic algorithm; (3) annotating IS with genomic features such as the nearest gene; (4) introducing an intuitive and user-friendly web interface to facilitate the usability of the tool; (5) improving the time speedup through highest parallelization of all steps (Hadoop free).

In this work, we describe the design and implementation of VISPA2, showing its performances both in terms of computational requirements and statistical assessment of precision and recall. Finally, we developed a user-friendly web interface to ease the usage of the tool.

## Implementation

### Bioinformatics pipeline

**Fig. 1:**
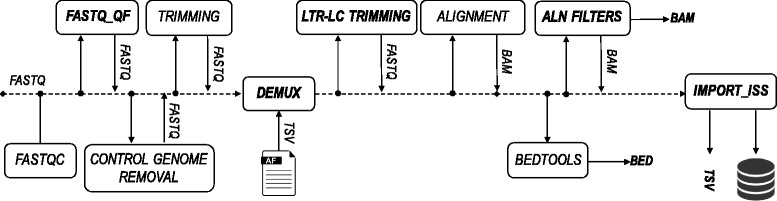
Workflow of the VISPA2 pipeline. The whole analysis process, starting from raw FASTQ to the final IS identification, in bold custom software

VISPA2 is a pipeline composed of several sequential steps that, starting from paired-end raw sequencing reads, generates the list of IS with genomic annotations (Fig. 1). In the first step *FASTQC* (Fig. 1), VISPA2 checks raw reads’ quality using FastQC [28] and filters out bad quality sequences (*FASTQ_QF*) below the threshold of Phred scale 15 (corresponding to 96.8%, Additional file 1, section 1). Adapters and internal control sequences are removed in the step named (*CONTROL GENOME REMOVAL*, *TRIMMING*). The remaining reads are then parsed within the *DEMUX* step to split reads into sample-specific FASTQ files identified by the designed tags (sample demultiplexing, Additional file 1, section 2 and 3). Long Terminal Repeat (LTR, the vector sequence flanking the cellular genomic junction) and Linker Cassette (LC, a synthetic DNA sequence attached to the fragmented genomic DNA) sequences are subsequently trimmed from each read to isolate only the genomic portion *LTR-LC TRIMMING*, and reads without LTR are discarded (Additional file 1, section 4). The remaining reads are mapped on the reference genome and the returned alignments are then scanned by the *ALN FILTERS* to avoid ambiguous alignments. All the IS are recorded in a structured file and optionally imported in a relational database for data mining purpose and easier data access and storage *IMPORT_ISS*. In a subsequent post-processing step, each IS is associated with the metadata of the source tissue sample from which it was originally derived (for example, peripheral blood or bone marrow), the cell type (for example, CD34, lymphoid T or B cells) and time point after treatment. Combining sample metadata with genomic information will allow to integrate data and perform IS data mining and other analyses.

Here we will describe the novel features of VISPA2, placing in Additional file 1 the description of the remaining steps.

### Alignment

After sequence quality filtering of sequencing reads, VISPA2 exploits publically available genomic alignment tools to find the exact location where the vector is integrated into the reference genome. VISPA2 can perform the IS analyses on any chosen reference genome by linking the selected genome to the setup configuration. The human reference genomes (hg19 GRCh37 and hg38 GRCh38) and the mouse reference genomes (mm9 and mm10) are embedded both in the online version of the tool and in the command line release for which we provide an automated configuration script. Different reference genomes or versions can be installed following the instructions in the Wiki page of the repository. We chose BWA-MEM [29] (with maximal exact match algorithm), thanks to its better performance compared to BWA-ALN or Bowtie2 [30, 31]. The alignment is configured with stringent parameters (Additional file 1, section 5) to best search for unique match on the target genome and with a minimum read length of 15 nucleotides.

After the alignment with BWA-MEM using both read pairs (in our experimental designs the R1 read contains the LTR and thus the IS genomic junction, whereas the R2 reads contain the LC ligated to the cellular genomic DNA end) VISPA2 processes the alignments using *SAMtools* [32]. For the alignment, we used stringent parameters of search and exploited the minimum read length at 15 for mapping. For the filtering procedure, we configured *SAMtools* to remove alignment with non-properly paired reads and with low quality alignment scores (mapping score of 12 in Phred scale), and non-primary alignments (see details in Additional file 1, section 5).

### Filtering

Since good quality alignments may present gaps or soft-clip at the beginning of the sequence and may have secondary alignments with similar scores to the primary alignment, we decided to further filter the mapping results using of two different steps: (1) *filtering by MATE* sequences and (2) *filtering by CIGAR* (Concise Idiosyncratic Gapped Alignment Report). Both these steps required the development of new algorithms and custom programs in Python.

#### Filter aligned reads by mate pair properties

The alignment of paired-end reads requires that mate reads are properly paired, meaning that R1 and R2 align in opposite orientations and with the last portion of the reads close to each other. In case of short DNA sequences, both paired-end reads may partially or entirely overlap, while, when longer DNA fragments are sequenced, pair-ends do not overlap and the distance between the two reads is called insert size.

In IS studies, the portion containing the LTR is crucial for the correct identification of the vector cellular genomic junction. For this reason, we imposed specific constraints to avoid wrong read trimming and consequently mapping errors. Since false positive IS can be generated by wrong trimming resulting in imprecise alignments, we designed the following rules to be satisfied by each aligned read to be considered a true IS:

1. Reads are properly paired.
2. If the DNA fragment is short enough to be sequenced from both ends, the alignment of the genomic portion is considered as proper, following these rules:
  - R2 must not end beyond R1 alignment start.
  - If R1 alignment ends exactly at R2 alignment start, then R2 end must be in the same position of R1 start (the case of fully overlapping and identical sequences).
  - If R2 and R1 are fully overlapping (only in this case), they should have the same the alignment score (unless a tolerance threshold of 5%). This filter is not applied when R2 and R1 are partially overlapping (which will be then used for the next steps of the pipeline).

If a read does not follow one or more of the rules is discarded. Since no existing tools can analyze mate properties applying these requirements, we designed a new command-line program to implement these specific rules. The program, *filter_by_mate*, has been developed in Python using the library PySAM [32], a package to process BAM (Binary Alignment/Mapping) files. To speed-up the performances, we parallelized the genomic selection (both whole chromosomes or specific regions that users can specify) such that each genomic region is processed as an independent process.

#### Alignment quality filtering by CIGAR and MD flags

The alignment quality of the sequencing reads can be inspected by their properties, which are generated and embedded by the aligner as optional flags of the BAM file format. The BWA-MEM algorithm [31] fills standard mandatory flag fields for alignment quality such as the CIGAR, MD (mismatching positions/bases), AS (alignment score) and XS (secondary alignment score). Since the MD field is a detailed description of the mismatches reported in the CIGAR flag, the combined usage of MD and CIGAR tags allows to better characterize the mismatches and base changes (insertions and deletions) of each sequencing read.

Given that IS with any mismatches in the first 3 bp may arise from PCR artifacts or wrong trimming of the LTR portion we analyze the beginning of the alignment and remove reads with mismatches, insertions, deletions or soft clipped alignments within the first 3 bp. We implemented this rule in the tool using the option *--minStartingMatches* (having default at 3).

We required that alignments were unequivocally mapped to the genome, without any alternative alignment in the genome that may suggest an IS landing in a repeated genomic region. To satisfy this goal, we replicated the rule applied in VISPA [22] by exploiting the BAM flags to remove aligned reads if the distance (δ) between the first (best) and the second alignment scores is lower than a threshold (corresponding to the program option *--suboptimaThreshold*), where the distance is computed as

$$ \delta =\left(1-\frac{XS}{AS}\right)\times 100 $$

In case of using a different aligner than BWA-MEM, users may configure the name of the flags with the proper option: *--ASlikeTag* and *--XSlikeTag*.

As an example, a read alignment having AS = 100 and XS = 80, will have δ = 20, thus using *suboptimaThreshold* > 20 will filter the read. The default value we provided is δ = 40 (see Additional file 1, section 6, for details).

We developed an ad hoc program for data filtering based on the evaluation of CIGAR and MD scores that applies the new rules that we could not retrieve by other NGS tools. We implemented the rules in a Python program called *filter_by_cigar_bam* that exploits the PySAM library to read input BAM files (creating the index if missing), splits the execution into independent processes based on chromosomes and processes reads by flags.

### Integration site data collection

Sequencing reads passing all filters are collected in a relational database and in a structured file reflecting the database table (for column order and content specification see Additional file 1 section 7 for database design and file structure). Moreover, during each step of the pipeline, VISPA2 collects in a table the number of reads passing the filter and discarded reads for each step by querying BAM files. These values are used to detect potential pitfalls along the pipeline processes and could be used for descriptive statistics and assessment of pool quality.

### Heuristic integration site merging

After being processed by the pipeline, IS data are acquired and structured as *covered bases* (or putative IS) that are the genomic coordinates of the bases mapping at the vector-host genomic junction.

Besides the genomic coordinate, each covered base has additional attributes such as the sequence count, a sequence header and the name of sample in which the IS has been retrieved (see Additional file 1, section 8 for further details).

Here, we define as *ensembles* the set of putative IS not far enough to be considered independent, and they may be the result of a dispersion effect of sequences stemmed from one or few IS. To generate the list of ensembles we developed an algorithm that scans the genome from the start to the end: when it encounters the first covered base by a sequencing read (putative IS), the first *ensemble* is instantiated. If the next covered base is less then Δ nucleotides apart, it is included in the current *ensemble*, and such rule is applied as long as the next covered base is more than Δ nucleotides away or if the chromosome ends. Under these circumstances the current ensemble is truncated and another one is instantiated. This procedure is repeated until all the covered bases have been exhausted and properly grouped in ensembles, even trivial ones (singletons). Moreover, covered bases in different ensembles are supposed to be related to different IS, for this reason, Δ is called interaction-limit, and is a parameter of our implementation.

Once all ensembles have been defined, they undergo to:

1. *Exploration*, detecting the local sequence count peaks within each ensemble.
  This step incrementally detects local sequence count peaks in a top-down fashion and, for each of them, the algorithm focuses on a sub-group of covered bases spanning at most (2*Δ+1) bp. The exploration is repeated until all the local peaks have been processed and linked to their sub-groups of neighboring covered bases. Notice that this is a redundant process because some covered bases in the ensemble may be included in more than a sub-group during this step, since the distance between two local flanking peaks, of ensemble, is less than Δ base-pairs.
2. *Evaluation*, quantifying the sequence coverage of all the covered bases surrounding each peak. This process involves all the peaks and related sub-groups of covered bases, assigning a score to each covered base with respect to the peak: the scoring procedure is based on the difference between each dispersion profile given as input with respect to the observed one (normalized histogram of sequence counts). At the end of this step, each covered base is scored as many times as the number of sub-groups it is included in during the exploration phase. Multiple-scored covered bases are conflicted bases between flanking peaks whose assignment will be solved in the decision step, since local peaks are supposed to be hallmarks of real and independent integration events.
3. *Decision*, identifying IS at the peak and assigning the surrounding bases.
  This step re-processes all the covered bases bottom-up, from the one with the lowest sequence count (to be reassigned) to the highest (underlining the more reliable IS positions). The algorithm assigns each covered base to a specific peak and, if this peak then is a covered base scored as belonging to another higher peak, it is absorbed along with its covered base cohort. At the end of the process each peak that is not reassigned by the algorithm is collapsed into a unique IS along with its cohort of related covered bases.

The pseudo-code for the Heuristic integration site merging is the following:
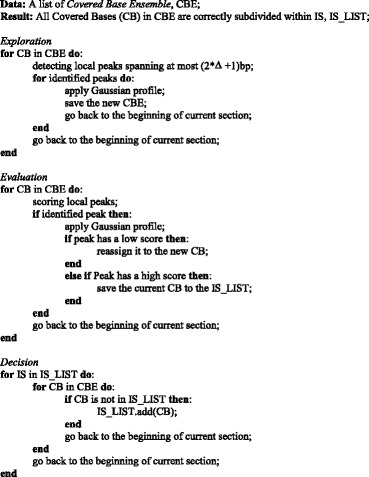


#### Implementation and tuning

We wrote this algorithm in Python programming language in order to generate a table of covered bases (rows) demultiplexed in samples (columns).

To maximize the flexibility of parameter tuning, our implementation allows the customization of Δ as well as the dispersion/ranking profile. Since the exploratory >analysis we made on large IS datasets did not suggest any particular profile and since single integration events have not been characterized through a distribution family yet in literature, at best of our knowledge, in this work we set a discrete Gaussian as the scoring profile, in order to avoid any a priori assumption and preserving the maximum generality. On this Gaussian curve, given that the μ parameter is set time by time by the algorithm as the locus of the peak, we can easily exploit Δ (set to 4 as noted in literature [33, 34]), to make its support finite (2*Δ+1) and choose σ in order to concentrate the 99.99% of the probability into such support, resulting in a profile fully determined. Eventually, we also added a default behavior such as the two adjacent bases to each identified peak are immediately assigned to it without any evaluation or choice, so that the minimum distance between two consecutive peaks is 2 bp. This is a workaround aimed at avoiding an empirically observed IS over-splitting, mainly caused by sequence count ties, with a consequent overestimation of putative IS.

### IS annotation

The final step is the IS annotation, in which each site is associated to the nearest genomic feature/s such as genes and potentially other annotations. For this task, we developed an annotation tool for the nearest genes called *annotate_matrix* (see Additional file 1, section 9).

For each IS, the program finds the closest gene among those listed in the annotation file and provides: the chromosomal coordinate and the orientation of each IS; the symbol of the nearest annotated RefSeq gene and the gene strand.

## Results

VISPA2 has been conceived to overcome computational limitations in IS studies and improve the accuracy of IS identification, in fields such as GT where the need for accurate and scalable computational tools is becoming everyday more demanding thus resulting a turning point for effective IS analysis and clinical trial monitoring to support the assessment of safety and long-term efficacy of the treatment.

In the continuous effort to improve the reliability of IS analysis, we developed VISPA2, a computational pipeline for IS mapping and analysis that contains several improvements with respect other available tools. The process of IS identification (Fig. 1) requires a workflow of several computational steps, from the quality inspection and filtering to the improved and optimized algorithms, which resulted not only accurate and reliable with respect to precision and recall assessment, but also with enhanced speedup in terms of computational performances.

The rigorous mapping of vector IS on the reference genome is critical and, since sequencing errors and/or PCR artifacts could potentially produce false positives. For this reason, we designed a new filtering tool based on the evaluation of BAM tags like CIGAR and MD to remove IS with poor quality alignments. Moreover, as reported previously [33], when aligning to the reference genome a large number of sequencing reads originating from the same IS some may align in slightly different positions wobbling around the true IS.

**Fig. 2:**
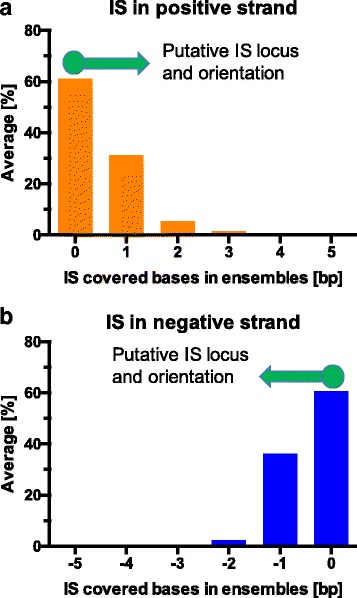
IS distribution downstream the LTR used for the PCR amplification with decreasing sequence counts. Clusters of putative vector IS were grouped in ensembles as described in material and methods. The abundance of the relative percent in sequence count of putative IS in each different position of each ensemble was calculated as the average of the relative percentage of sequence count for each putative IS on the total reads associated to each ensemble. Downstream vector IS the abundance is relatively high and decreases progressively with the distance. Both the asymmetric distribution with respect the LTR orientation and the gradual decrease in abundance in function of the distance in forward orientation (**a**) and reverse orientation (**b**) indicate that these putative IS are false positives

In the dataset of 21,895 putative IS retrieved from a gene therapy patient [35], 10,475 (48%) were in a single position without neighboring IS. The remaining putative IS, having at least another neighboring IS were grouped in 4122 ensembles among which 199 were constituted by 2 to 4 putative IS with a size between 3 and 18 bp. All putative IS of each ensemble aligned on genome with the same orientation and a marked tendency of the putative IS with the highest sequence count to cumulate on one side of the ensemble interval while the remaining putative IS had progressively decreasing sequence counts across the interval (Fig. 2a–b). The strong bias in the distribution of the putative IS in terms of orientation and sequence count, was a clear indication that the putative IS were false positives, likely generated by the presence of nucleotide variations with respect to the reference genome, or as a consequence of PCR artifacts, sequencing or trimming errors.

**Fig. 3:**
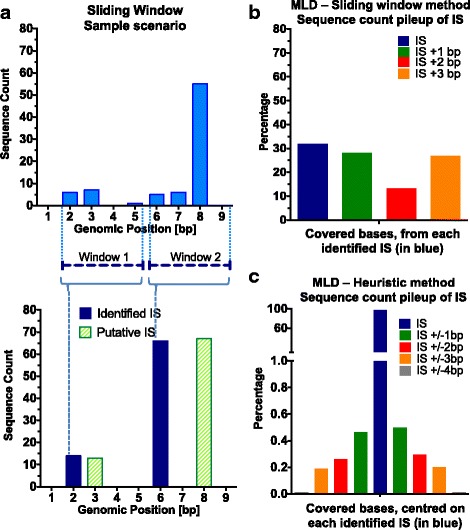
Sliding Window and Heuristic Method applied on MLD patients. **a** Sliding window approach with a sample scenario highlighting a methodological limitation in terms of precision. The upper graph presents a scenario of covered bases in the genome (x-axis) with their sequence count (y-axis) where the first covered base is in position 2. The SW method applies two windows in the interval 2–5 bp, and 6–9 bp, resulting in the bottom histogram plot as two IS, placed in position 2 and 6 respectively (blue bars) and with sequence count derived from the sum of all the sequence counts of the covered bases belonging to its own window. Putative IS positions and heights are represented with green histograms. **b** Bar plot of relative percent of sequence count of putative IS within the window span of 4 bp by the sliding window approach. The blue bar is in the first position, the putative identified IS, whereas the other bars represent the IS mapping in the neighboring bases within the same window at a distance <4 bp from the first ensemble base. **c** Heuristic approach applied to MLD patients. The bar plot represents the relative percentage of sequence counts for all putative IS in the interval +/− 4 bp from the base with the maximum sequence count, the putative output IS (blue bar) and the distance of the other IS in the same interval from it

To eliminate this type of false positive IS, previous studies have exploited an approach consisting of a rigid sliding window (SW) of 4 bp [33, 34], where all putative IS within an interval of four nucleotides are merged to the same putative IS at the first base of the window, and their sequence count added to the count of the first putative IS without considering the read distribution, peak locations, or any statistical consideration about artifacts. If the ensemble (a cluster of putative IS) spans more than 4 bp the SW will move to the next 4 bp and create another IS as described above. Assuming that the true IS should have the highest sequence count when compared to the neighboring false positive IS, the sliding window method could misplace the true IS (Fig. 3a). Analyzing with the SW method a dataset of 54,309 putative IS retrieved from three patients of a HSC GT clinical trial [35], the distribution of the sequence counts of SW of 4 bp did not show a clear peak at the identified IS at the first position that is considered to be the true IS (Fig. 3b). This is mainly caused by the lack of considering the orientation of the IS in the window and the lack of centering the IS on the sequence count peak. To solve this issue, we developed a heuristic method that merges false nearby IS that leverages on a proximity criterion to partition the genome into uncorrelated regions and then, for each of them, it explores the local sequence count peaks, ranking its surrounding reads exploiting a user-defined dispersion profile, and lastly condensing data in one IS. When the same IS dataset from MLD patients was reprocessed by the heuristic-based algorithm, we analyzed the distribution of the putative IS (Fig. 3c) that showed a symmetric profile on the centered base of IS with the highest sequence count.

**Fig. 4:**
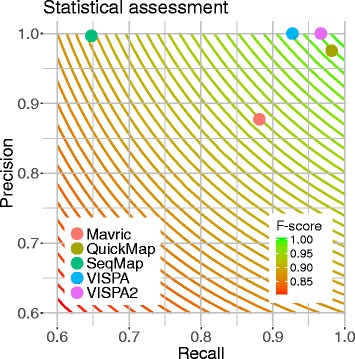
VISPA2: Precision and Recall. Precision and recall of all the tested tools Mavric, SeqMap, QuickMap, VISPA and VISPA2. Rounded curves are the F-score levels, with color code green at value 1 to red at value 0.8

To assess precision and recall performances of VISPA2, we used the simulated dataset of 455 IS already used for the validation of the previous pipeline VISPA [22] and here we considered true positive values (TP) all IS returned as valid IS and having the same genomic position within a range of 3 bp, false positive IS (FP) all IS with wrong genomic coordinates, and false negative IS (FN) all IS not returned. Under this setting, VISPA2 was able to correctly identify 440 IS (TP, 98.9%) and no FP, and 15 FN. We then run the simulations on other available tools for IS identification such as VISPA [22], Mavric [16], SeqMap [14] and QuickMap [15] and we evaluated their performances (Table 1, Additional file 2). VISPA2 showed a precision and recall of 1.0 and 0.97 respectively, a clear improvement with respect to VISPA [6], Mavric [9], and SeqMap [7] (Fig. 4). QuickMap [8] provided comparable results although false the positives reached 2.4% of the total (see Additional file 2) but reached a lower F-score than VISPA2 (QuickMap F-score 0.978, VISPA2 F-score 0.983). The statistical assessment thus showed the performance improvements of VISPA2 in terms of precision and recall.

**Table 1:** Comparative results of simulated IS obtained from different tools

	VISPA2	VISPA	MAVRIC	SeqMap	QUICKMAP
TP	440	422	357	294	436
FP	0	0	50	1	11
FN	15	33	48	160	8

A dataset of 455 simulated IS generated previously [22] was used to test the performance of VISPA2 and other available IS mapping tools. In the confusion matrix used to assess precision and recall we defined: *TP* True Positives, number of IS correctly mapped into the genome (with a tolerance of 3 bp); *FP* False Positives, number of IS mapped in a wrong genomic location (>3 bp from the theoretical locus); *FN* False Negatives, number of discarded IS

### Computational improvements

**Fig. 5:**
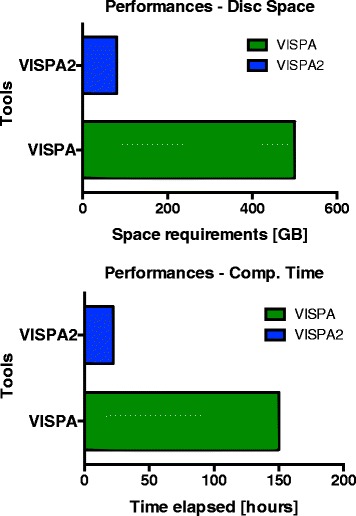
VISPA2 Performances. VISPA2 Performances compared to VISPA. The test, with an Illumina HiSeq run (186,300,301 of reads), revealed the improvements of VISPA2 in term of performances in space (**a**) and time (**b**) required to accomplish the task

We assessed the improvements of VISPA2 in terms of computational time and space by comparing results of performances against VISPA (that was the fastest tool compared to Mavric, SeqMap and QuickMap, as reported in [6]). We used two types of Illumina sequencing runs, a MiSeq run of 14,583,450 reads (2.5GB FASTQ compressed) and a HiSeq run of 186,300,301 reads (20GB FASTQ compressed). The resulting space and time required to process each of the two NGS runs showed an increase of 6/7-fold (respectively) for VISPA2 with respect to VISPA (Fig. 5). For example, for the HiSeq sequencing run VISPA2 took 75GB of disk space, instead of the 500GB of VISPA, whereas VISPA2 completed the task in 23 h, instead of the 150 h of VISPA.

### Software release

We released VISPA2 both as a web tool (for demo purposes) and command line version (for large computational requirements). Both versions implement the same features of VISPA2, from the type of input files (single or paired end reads) to the output annotated results.

VISPA2 web site is freely accessible at the URL http://openserver.itb.cnr.it/vispa. The web application was developed using Java and Javascript technologies in JSP pages. User manuals are available in the source repository as Wiki pages and in the web site. Moreover, we provide an automated setup/installer script to facilitate user installation, interaction and configuration of the tool.

**Fig. 6:**
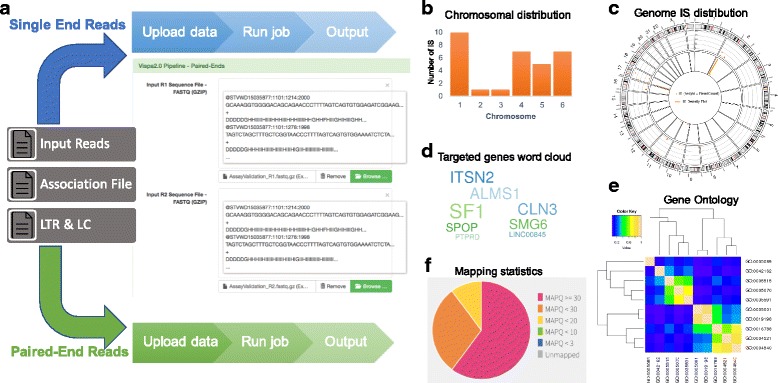
Web Interface, workflow. A web version of VISPA2 is freely available at http://openserver.itb.cnr.it/vispa, it is open to all users and no login required, although there is a 50 MB limit in the size of input data (for larger analysis, please download the pipeline on your server or contact the authors). The figure shows a flowchart of the application. **a** At the first page the user can specify to run the single-end version or the paired-end version of the pipeline. In a second screen the user must upload the input sequences (demo examples are also provided) and set the VISPA2 parameters. Clicking for the next page, data are uploaded to the backend server. Then, a submission page is presented to the user that must confirm all the information provided. Clicking for the next page, the computation starts. At this point, a results page is presented, which shows the pipeline advancement while the computation is running. The user can wait for the end of the computation or bookmark this address and return later. Once the pipeline is finished, the same page presents the results achieved by the VISPA2 pipeline. **b**–**f** In the results page, different statistics are reported (the output is the same for the single-end and the paired-end version): (**b**) a histogram of the IS distribution in the genome is shown, while in the bottom part some tab panels are present, showing different detailed statistics. The first tab contains a table showing the specific chromosome locus and strand of each IS, also reporting the nearest gene. The second tab (**c**) presents a circos plot of the IS density in the genome, while (**d**) a tag cloud of the genes more targeted by insertions is plotted in the third tab. We also implemented a Gene Ontology (GO) enrichment analysis of the target genes (**e**), considering the three branches of GO (Molecular Function, Biological Process, and Cellular Components), which is shown in the fourth tab. Beside the *p*-values achieved in this analysis, a diagram is reported of the most representative GO terms, bi-clustered according to their semantic similarity. The last tab (**f**) represents the statistics concerning the dataset computed by *samstats* [38]

The main flow of the web application, from the first user access is represented in Fig. 6a. A welcome page introduces user to VISPA2 and presents the possibility to select the pipeline that best fits input reads: single or paired-end reads. In both cases the user can upload a FASTQ file with the sequencing reads (compressed file with GZIP or plain; FASTA file format is accepted only in single read mode so that users can use VISPA2 with file of sequences without per-base quality information) and a metadata file, created with adLIMS [36], where each row contains information of the corresponding sample included in the input FASTQ file associated to sequencing reads by barcodes (attached to LTR and LC). The web interface also presents all available options to parametrize the pipeline for custom experimental designs (for example different LTR or LC sequences) or to change the default parameters, here optimized for the standard experimental protocols [25, 37]. A full working example is uploaded by default (see wiki in the repository for details). Once configured the run and clicked the start button, the web interface presents a summary page in which the user can check all the parameters, and, once approved, the computation job is started. The job could last several minutes depending on the input file size, and, after completing the task, VISPA2 shows a result page whose link and job ID can be saved and viewed later.

The output page (Fig. 6b–f) shows both a summary of the results, tabs that enable browsing different sets of results (according to input metadata), and the resulting comprehensive IS matrix. This matrix has a column for each dataset and a row for each IS, and cells contain the number of reads mapping to that IS (the meaning of zero is missing value/observation for that dataset). The result page also presents different statistics and analyses for each of the computed datasets. In the upper part of the page, VISPA2 summarizes the IS distribution in the chromosomes with a histogram (Fig. 6b), while in the bottom part different tab panels present different statistics. The first tab reports a table showing for each IS the targeted chromosome locus and strand, and the nearest gene. User can also export results in this matrix file format for user analysis. The second tab shows a circus-plot of the IS density in the genome to understand potential skewing of genes in specific genomic regions (Fig. 6c), while in the third tab the top targeted genes by IS are visualized in word-cloud representation (Fig. 6d). The fourth tab shows the results of Gene Ontology (GO) enrichment analysis of the targeted genes (Fig. 6e), considering the three branches of GO (Molecular Function, Biological Process, and Cellular Components). These results are useful for understanding potential enrichment in gene classes related to cancer or tumor development. Beside the *p*-values achieved in this analysis, a diagram is reported of the most representative GO terms, bi-clustered according to their semantic similarity. The last tab presents the statistics concerning the dataset computed by the integrated tool *samstats* [38] as per base alignment report of IS sequencing reads (Fig. 6f).

## Conclusions

Bioinformatics pipelines for IS analysis have been specifically designed to analyze DNA fragments generated using specialized PCR protocols able to amplify DNA fragments containing the junctions between the integrated vector and the cellular genome [23]. Thus, sequencing and mapping of these PCR fragments allows to localize IS in the reference genome. However, these PCR products contain not only the cellular genomic sequence but also viral and artificial sequences that must be trimmed out before alignment to the reference genome. Moreover, sequencing reads must be processed by a bioinformatics pipeline that yields not only the list of the genomic coordinates of each IS but also a set of genomic annotations, such as the nearest gene, important for the evaluation of the safety of vector integration in preclinical and clinical gene therapy applications. VISPA2 was designed to reduce the time and space requirements (fully compliant to paired end reads) and increase the accuracy of IS identification. To fulfill these goals, we introduced and developed new features: (1) paired-end reads support to manage DNA fragmentation methods based on sonication for IS retrieval as applied to Linker-Mediated-PCR [25, 26], (2) quality filters both on the input raw reads, reducing false positive IS calling, and on aligned reads using the CIGAR and MD tags, (3) a module to better distinguish between nearby IS using a heuristic algorithm, All steps have been implemented fully parallelized, achieving a > 6-fold in speed and >7-fold reduction in space required for the analysis with respect to our previous tool. We also developed and released a web interface to freely access the demo version of the tool.

These upgrades, combined with a high scalability, allow VISPA2 to be used in long term gene therapy applications, as needed when starting a clinical trial and in the context of the commercialization of gene therapy treatments [39].

## Additional files

Additional file 1: Supplementary Information. Supplementary Material, Figures and Tables. (DOCX 1859 kb)

Additional file 2: In silico dataset and accuracy assessment results. The excel table reports the list of all IS (in rows) and the corresponding output returned by the different tools (divided by colors in the following order: VISPA, VISPA2, MAVRIC, SEQMAP, QUICKMAP). For each read (identified by its “ID” in column “header”), we reported the source genomic coordinates (in columns chromosome “chr”, integration point “locus”, and orientation “strand”), the source of annotation as described in VISPA [22] and the nucleotide sequence. Then we reported the output of IS for each tool: the first set of columns report the returned IS genomic coordinates (columns “header”, “chr”, “locus” and “strand”), whereas the other columns label each IS for statistical assessment as true positive (TP), false positive (FP), and false negative (FN) based on the genomic distance (“IS distance”) from the ground truth. Precision and recall are then derived by the columns of TP, FP, and FN. (XLSX 233 kb)

## Abbreviations

AS: Alignment score
BAM: Binary alignment/mapping
BWA: Burrows-Wheeler Aligner
BWA-ALN: BWA Aligner
BWA-MEM: BWA Maximal Exact Match
CB: Covered bases
CBE: Covered Base Ensemble
CIGAR: Concise Idiosyncratic Gapped Alignment Report
DNA: Deoxyribonucleic Acid
FN: False Negatives
FP: False Positives
GO: Gene Ontology
GT: Gene therapy
hg19: Human genome build 19
hg38: Human genome build 38
HIV-1: Human Immunodeficiency Virus
HSC: Hematopoietic stem cell
HTLV-1: Human T-Lymphotropic Virus
IS: Integration Sites
LC: Linker Cassette
LTR: Long Terminal Repeat
MD: Mismatching positions/bases
mm10: *Mus Musculus* genome build 10
mm9: *Mus Musculus* genome build 9
NGS: Next-Generation Sequencing
PCR: Polymerase Chain Reaction
R1: Read 1 (5′-3′) in paired-end mode
R2: Read 2 (3′-5′) in paired-end mode
SW: Sliding window
TP: True Positives
XS: Secondary alignment score
