# Supplementary material for: VISPA2: a scalable pipeline for high-throughput identification and annotation of vector integration sites
Source: BMC Bioinformatics. 2017 Nov 25;18:520. doi: 10.1186/s12859-017-1937-9 (PMC5702242; doi:10.1186/s12859-017-1937-9)
Supplement: Supplementary file 1 — Supplementary Information. Supplementary Material, Figures and Tables. (DOCX 1859 kb) [file 12859_2017_1937_MOESM1_ESM.docx]

SUPPLEMENTARY INFORMATION

**VISPA2: A Scalable Pipeline for High-Throughput Identification and Annotation of Vector Integration Sites**

Giulio Spinozzi^1, †^, Andrea Calabria^1, †^, Stefano Brasca^1^, Stefano Beretta^2^, Ivan Merelli^3^, Luciano Milanesi^3^ and Eugenio Montini^1, §^

^1^San Raffaele Telethon Institute for Gene Therapy, Safety of Gene Therapy and Insertional Mutagenesis Research Unit, Via Olgettina, 58, 20132, Milan, Italy

^2^University of Milano Bicocca, Department of Computer Science, Viale Sarca, 336, 20126, Milan, Italy

^3^National Research Council, Institute for Biomedical Technologies, Via Fratelli Cervi, 93, 20090 Segrate, Italy.

^†^ Contributed equally to this work

^§^ Corresponding author

Email Addresses:

GS: spinozzi.giulio@hsr.it

AC: calabria.andrea@hsr.it

SBra: brasca.stefano@hsr.it

SBer: beretta@disco.unimib.it

IM: ivan.merelli@itb.cnr.it

LM: luciano.milanesi@itb.cnr.it

EM: montini.eugenio@hsr.it

**Keywords**

Open Source Software, Bioinformatics Pipeline, Integration Site Analysis, Gene Therapy, High-Throughput sequencing

Table Of contents

[1. Quality Controls and Filters 3](#_Toc494462991)

[1.1 Quality check 3](#_Toc494462992)

[1.2 Filter Raw Reads by Sequencing Quality 3](#_Toc494462993)

[2. PhiX removal and random barcode trimming 6](#_Toc494462994)

[3. Demultiplexing and sample association file 7](#_Toc494462995)

[4. LTR/LC Trimming and Internal Control Removal 8](#_Toc494462996)

[4.1 Identification and filtering of vector-only reads 10](#_Toc494462997)

[5. Alignment to Reference Genome 11](#_Toc494462998)

[6. Filtering by CIGAR and MD flags 12](#_Toc494462999)

[7. Integration Site Import in MySQL Database and Stats Summary 13](#_Toc494463000)

[7.1 Integration Site Table 13](#_Toc494463001)

[7.2 Integration Site _refactored Table 13](#_Toc494463002)

[7.3 Stats Summary 14](#_Toc494463003)

[8. Heuristic Integration Site Merging 16](#_Toc494463004)

[9. Integration Site Annotation 18](#_Toc494463005)

[References 19](#_Toc494463006)

# Quality Controls and Filters

Raw data quality controls are required to remove false positive IS generated by PCR artefacts or sequencing errors. We first check for raw data and then we filter data by quality.

## Quality check

VISPA2 checks on raw sequence data generated by high throughput sequencing with *FastQC* [1]. This allows to perform quality control checks on FASTQ files. To filter out reads with low quality we created a custom quality filter specific to analyze sequences of LAM or Linker-Mediated-PCR [2] products. The bash script to execute the program is: **fastq_qf**, *VISPA2/script/fastq_qf.sh*.

## Filter Raw Reads by Sequencing Quality

The sequence quality filter is applied on the first 80bp from R1. This window is created with a parallel trimming tool (to improve performances) that is *Trimmomatic* [3] and *fastq_quality_filter* a part of FASTX-toolkit (<http://hannonlab.cshl.edu/fastx_toolkit/index.html>), adopting an optimal set of parameters (-q 28, -p 95, -Q 33). Then the program extracts only the high-quality reads with **fqextract_pureheader** (*VISPA2/script/fqextract.pureheader.py*).

For PCR methods for IS retrieval that use sonication to fragment the DNA, an additional sequence quality filter is extended to the random barcode of 12bp on the linker cassette from R2 pair, which is fundamental for quantification purposes. The optimal set of parameters for *fastq_quality_filter* on this relatively short sequence: -q 28, -p 100 and -Q 33. Then two lists for high quality reads for the 80bp of R1 and the12bp of R2 are created and merged (with *comm* command in bash). The final FASTQ file will contain only sequences for which the two pairs (R1 and R2) passed the quality filter.

**fastq_qf**, *isatk/script/fastq_qf.sh* gives in input only 5 parameters:

1. **[-a** R1.FASTQ.GZ] - Input File: Illumina R1 FASTQ zipped.
2. [**-b** R2.FASTQ.GZ] - Input File: Illumina R2 FASTQ zipped.
3. [**-o** output dir] - Output Directory.
4. [**-t** max threads] - Maximum Number of Parallel Threads.
5. [**-m** method] - Quality Filter on R1 only (LAM) or R1 and R2 (Linker-Mediated-PCR).

To test the goodness of our quality filters we tested two different Illumina sequencing runs of LAM PCR products from the same DNA sample form a human cell line transduced with a lentiviral vector (checked with *FastQC*) with bad (ET6, median base quality <28) or good sequence quality (ET6v2, median base quality ~38) (**Figures S1, S2)**.


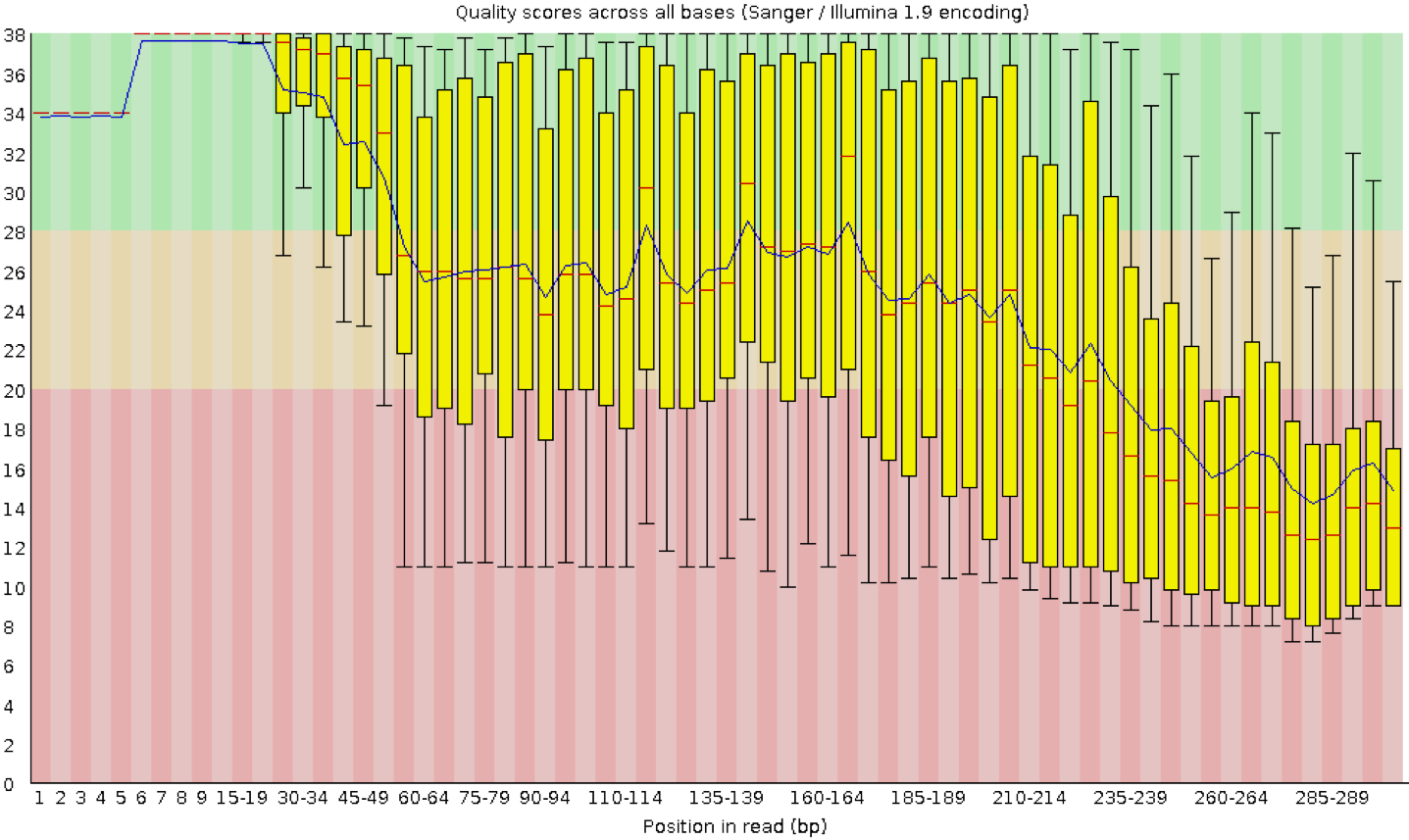


**Figure S1**: Per base sequence content for sequencing pool ET06 obtained with FastQC. The plot shows a per-base sequence content where x-axis is the full length of a read (300bp), y-axis the distribution of the sequence quality (with median and quantile values in boxplots).


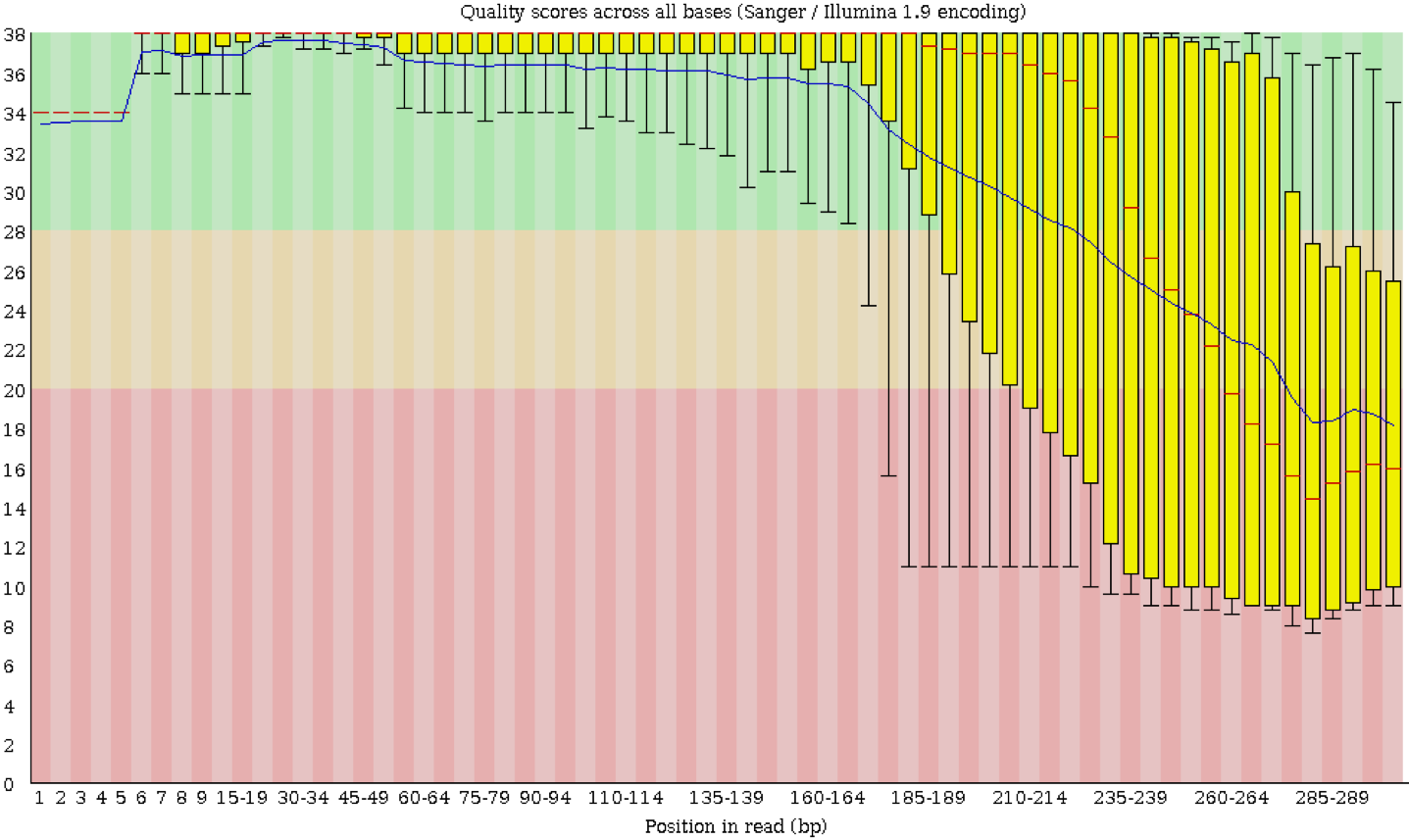


**Figure S2:** Per base sequence content for the re-sequenced pool ET06 obtained with FastQC. The plot shows a per-base sequence content where x-axis is the full length of a read (300bp), y-axis the distribution of the sequence quality (with median and quantile values in boxplots).

When the low-quality sequencing run ET6 was analyzed by our pipeline without quality filters yield 4,592 univocally mapped IS. Application of our filter on this low-quality sequencing run resulted in the complete elimination of all sequencing reads and consequently no IS were identified. On the other hand, when the high quality ET6v2 sequencing run was analyzed yield 4006 IS. Application of the sequence quality filter on this sequencing run (ET6v2HQ) reduced the number of IS to 3586 (10.48% reduction).

To test the relevance of the adoption of stringent sequence quality filters we compared the IS found in the ET6v2 and ET6v2HQ datasets. From this analysis, we observed that all IS of the ET6v2HQ dataset were contained in the ET6v2. Importantly, we found that 432 IS were present only in the ETv2 dataset.

# PhiX removal and random barcode trimming

Once filtered the reads by quality the next step is to remove the reads mapping in PhiX (a control for Illumina sequencing runs) and the first 12bp of random barcodes from R1.

VISPA2 removes PhiX sequences first by aligning all the reads on PhiX genome with BWA-MEM, producing a list of the reads that map on that genome and discard them using **fqextract_pureheader**.

The first 12bp of R1 are be removed from the read with *Trimmomatic* [3] (as described above). The first 12bp of R2 are removed from the read and saved in a FASTA file for quantification purposes. To improve the performances, in term of time, we selected to use parallelism in our computers.

# Demultiplexing and sample association file

Several samples are often sequenced at the same time, a strategy called multiplexing. To enable the redistribution of output reads into separate groups (demultiplexing), samples are tagged with individual 8bp tag sequences. We demultiplexed our samples with *fastq-multx*, a part of the *EA-Utils suite* (<http://code.google.com/p/ea-utils>). It identifies each sample-specific tag and uses them to demultiplex sequence data producing a separate FASTQ file for each tag. To avoid biases due to the possible misclassification of similar sequences, 1 mismatch is tolerated in this phase.

All information regarding samples are written in the AF. This file is created automatically from *adLIMS* [4]. The AF (tab-separated file) contains all the following fields, as reported in **Table S1**. The first two columns contain the barcodes list, for demultiplexing, the other fields all the metadata for IS analysis. Indeed, is reported the tissue of the sample, a sample ID, the time point of the harvest, the LAM PCR reaction ID (LAM-ID) for backlinks identification of the sample, a unique complete containing all required fields for MySQL non-blind identification of a specific sample, the cell marker, the enzyme used for the LAM PCR reaction (if Linker-Mediated-PCR this filed is unused) and finally the type of vector used.

| **Tag ID** | **Tissue** | **Sample** | **Time Point** | **LAM-ID** | **Complete Name** | | **Cell Marker** | **Enzyme** | **Vector** |
| --- | --- | --- | --- | --- | --- | --- | --- | --- | --- |
| LTR22.LC32 | PB | 03 | 06 | ET#13.1 | | 03_PB_MNC_06 | MNC | tsp | PGK.ARSA |
| LTR22.LC64 | PB | 03 | 06 | ET#13.2 | | 03_PB_MNC_06 | MNC | tsp | PGK.ARSA |
| LTR22.LC74 | PB | 03 | 12 | ET#13.3 | | 03_PB_MNC_12 | MNC | tsp | PGK.ARSA |
| LTR22.LC90 | BM | 03 | 12 | ET#13.4 | | 03_BM_CD34_12 | CD34 | tsp | PGK.ARSA |
| LTR60.LC32 | BM | 03 | 06 | ET#13.5 | | 03_BM_CD34_06 | CD34 | tsp | PGK.ARSA |

**Table S1:** example of association file in the first column Tag ID contains the IDs of the specific oligonucleotide and linker cassette combination (each having a known sequence tag of 8 nucleotides) used. Tissue: the tissue source used (PB: peripheral Blood, BM: Bone Marrow); Sample: name of the sample; Time point: time of harvest (in months) after gene therapy; LAM ID: ID of the Linear Amplification Mediated (LAM) PCR reaction; Complete Name: unique superstring ID generated by the concatenation of sample name, tissue, cell marker and time point; Cell marker: the cell type used (MNC: mononuclear cells, CD34: CD34+ surface marker) of the sample; Enzyme: the enzyme used for DNA digestion; Vector: vector ID used.

# LTR/LC Trimming and Internal Control Removal

After the demultiplexing, the TRIMMING step is aimed at removing vector sequences, such as LTR and LC, from input reads (both paired ends and single reads). Since the vector cellular genomic junction, that is the IS, is identified by the genomic sequence after the LTR, the recognition and removal of the LTR and LC is done with *FLEXBAR* [5]*{Dodt, 2012 #448}*, due to its option to search for the LTR sequence scanning the input read from the beginning (option “LEFT” with the same accuracy applied in VISPA trimming, that is minimum of 90% sequence homology), thus allowing to accept any type of LTR input sequence.

that enables accurate recognition, sorting and trimming of sequence tags with maximal flexibility. The software supports data formats from all current sequencing platforms, including paired-end reads, with multi-threading support.

Setting up the best parameter configuration to correctly identify IS from the beginning of each sequencing read, has been generated using a statistical framework that explored a wide set of configurations and selected the best solution by assessing precision, recall, specificity and sensitivity.

The statistical framework is composed by two main steps (**Figure S3**): LTR analysis and trimming calibration. For the LTR analysis we used a dataset of 20,000 sequencing reads of LM-PCR products generated by sonicated DNA of a human cell line transduced with a lentiviral vector. We thus inspected the nucleotide composition of the FASTQ focusing on the 32bp long LTR sequence downstream the oligonucleotide used for PCR amplification (ACCCTTTTAGTCAGTGTGGAAAATCTCTAGCA). *BWA-MEM* was used to align each sequencing read to the reference LTR and determine the base composition and all potential mutations (base change, insertions and deletions). To identify mutations, we used *SAMtools* for piling-up aligned reads and *Varscan2* (http://varscan.sourceforge.net) for variation calling. Once obtained the per-base sequence composition of the LTR (**Figure S3**), we used custom scripts to determine the alignment length for each LTR sequence identified (start and end of the alignment on the reference LTR), nucleotide variations (mutations or indels) and the number of sequences having the same LTR sequence. All LTR sequences are therefore represented as *multiple sequence alignment* (MSA) to better understand the empirical distribution of the different alignments and LTR variants in the sequencing pools (**Figure S3)**.

**Figure S3:** Workflow of the LTR trimming parameter setup. Procedure is split in two phases, “LTR analysis” and “Trimming calibration”. In the “LTR analysis”, we first align reads to the reference LTR sequence, then we piled-up the reads and find variations to plot in different plots: a barplot and a logo plot of the sequences along the reference LTR sequence, the multiple sequence alignment plot of empirical unique sequences and their abundance. Once created the simulated dataset using the percentages of the empirical cases, the second phase “Trimming calibration” can start and ends with an optimization procedure to evaluate the best parameter configuration using statistical assessment of precision and recall.

With this approach, we observed that about the 30% of the reads had an incomplete LTR sequence terminating after the oligonucleotide used for PCR amplification. This phenomenon is expected since DNA sonication will break the LTR in random positions. The reads showing different lengths of alignment on the reference LTR (from 32 to 28bp) were grouped in classes. Then we extracted the portion downstream the LTR sequence and calculated the information content of the DNA sequence for each alignment group.

The alignment groups with LTR alignments ending at 29bp or before were always fused to the sequence of the linker cassette. On the other hand, LTR alignment groups ending at 32, 31 and 30bp of the LTR were fused to highly different *bona fide* genomic sequences, without any peculiar sequence content bias. These considerations led us at the following conclusion: a raw read can be considered and labeled as putative IS if the LTR portion of the sequence ends from base 30 to the end, thus no more than 2 mismatches at the end part of the sequence.

To calibrate the *FLEXBAR* trimming parameters we attached to each of the 20,000 LTR sequences with different lengths (alignment groups) a specific genomic sequence (LTR + identical genomic region, chr1:21367764-21474858). This reference file was then processed using a wide set of parameter configurations and then assessed by statistical measures: sensitivity and specificity (**Figure S4**).

By this statistical assessment, we identified the best parameter configuration for *FLEXBAR*: -ae LEFT -m 18 -q 1 -ai -4 -ao 22 -at 3.6.

**Figure S4:** Statistical framework to evaluate the best software configuration. (**A**) Definition of the delta measure. Given a sequence composed by the LTR and the genomic portion, the junction point is the integration site. A trimming procedure may improperly cut the LTR and generate a delta of the putative IS, that is the distance in bp from the correct position. This distance is here called delta. (**B**) Statistical framework to evaluate the parameter configurations (TP: number of True Positives; FP: number of False Positives; FN: number of False Negatives; TN: number of True Negatives)

Users can define any LTR or LC sequences in a FASTA file that will be parsed by VISPA2.

## Identification and filtering of vector-only reads

Many of the PCR protocols for IS retrieval generate also a byproduct containing only vector sequences. To eliminate these non-informative sequences that theoretically amount to 50% of the entire dataset we align paired-end reads with *BWA-MEM* [6] to the lentiviral vector genome. The sequencing reads perfectly mapping on the vector genome are then discarded. Usually, 30% of the total sequencing reads are removed in this process.

# Alignment to Reference Genome

To find the exact location where the vector is integrated into the genome, sequencing reads must be mapped to a reference genome. VISPA2 gives to *BWA-MEM* the two pairs (R1 and R2) and then *SAMtools* [7], processed in the following way:

1. **bwa-mem** -k 18 -r 1 -M -T 15 -c 1 -R -t ${THREADS} ${R1.FASTQ} ${R2.FASTQ} > ${TMPDIR}/sam/SAMPLE_1.sam
   1. *-k* [18]: Minimum seed length. Matches shorter than *k* will be missed.
   2. *-r* [1]: Trigger re-seeding for a MEM longer than *minSeedLen*r*. This is a key heuristic parameter for tuning the performance. Larger value yields fewer seeds, which leads to faster alignment speed but lower accuracy.
   3. *-M*: Mark shorter split hits as secondary (for Picard compatibility).
   4. *-T* [15]: Do not output alignment with score lower than 15 (Phred Quality Score). This option only affects output.
   5. *-c* [1]: Discard a MEM if it has more than *c* occurrence in the genome. This is an insensitive parameter.
   6. *-R* [COMPLETE_NAME]: Complete read group header line. ‘\t’ can be used in STR and will be converted to a TAB in the output SAM. The read group ID will be attached to every read in the output. An example is ‘@RG\tID:foo\tSM:bar’.
2. **samtools** view -F 2308 -uS ${TMPDIR}/sam/SAMPLE_1.sam
   1. *-F* [FLAG]: Filter the alignments that will be included in the output to only those alignments that match certain criteria (<https://broadinstitute.github.io/picard/explain-flags.html>).
   2. *-uS*: Output uncompressed BAM. This option saves time spent on compression/decompression and is thus preferred when the output is piped to another *SAMtools* command. Ignored for compatibility with previous *SAMtools* versions. Previously this option was required if input was in SAM format, but now the correct format is automatically detected by examining the first few characters of input.

# Filtering by CIGAR and MD flags

We obtained the threshold of 40% by analyzing the distribution of the delta (δ) scores obtained from a dataset of IS from a gene therapy patient in which the IS post alignment showed also a second hit (excluding all sequences that mapped univocally on the genome). The distribution of the δ-scores of these IS (**Figure S5**) was multimodal, showing a peak at the lowest values of δ-score representing the contribution of sequences with low mappability where the scores of the first and second hit were almost identical (δ between 0 and 20, area under the curve AUC = 0.2158), a peak at the highest values of δ-score representing the contribution of sequences in which the second hit has a much lower value with respect the first hit; a relatively flat interval comprised between the two aforementioned peaks with intermediate δ-score values that represent the contribution of sequences with different levels of mappability (δ between 80 and 100, area under the curve AUC = 0.2063). The two peaks at the extremes of the distribution represent the sequences that are clearly repetitive or clearly unique. The portion comprised between these two peaks represents a “gray area” composed by sequences displaying a continuum from low to high mappability. Since the average of the δ distribution showed a value of ~40, we applied it as default value. However, depending on the specific applications, the selection of reads based on their mappability can be more or less stringent. For this reason, the threshold is not static and users can increase the stringency (>40) of relax the stringency (<40) by changing the *suboptimaThreshold* parameter, both in the web interface and in the command line version of VISPA2.

**Figure S5**. Density plot of the δ score for patient MLD01. The score ranges from 0 to 100, where 100 means that the best hit compared with the second hit is predominant (thus the read is comparable to an unambiguously mapped read) whereas 0 reports the reads with best hit and second hit almost identical (evidence of read mapping in a repetitive element). The yellow dashed line shows the δ value that we selected.

# Integration Site Import in MySQL Database and Stats Summary

Two programs (*VISPA2/script/ dbimport_redundantiss_from_bed.v2.py* and *VISPA2/script/ import_iss.p*y) take care of the filtered IS files and import the reads in a MySQL databases, in two different tables:

- The integration site table: it contains the minimum information set for IS reads like genomic coordinate and metadata.
- The integration site refactored table: it contains alignment information of the mapped IS reads acquired from BAM files.

The *stats summary* table is used by VISPA2 to acquire sample specific reads counts for each activity showed in the pipeline (**Figure 1**). These data are useful both while analyzing VISPA2 performances and for sample statistics.

## Integration Site Table

1. group_name: project name
2. n_LAM: LAM ID
3. pool: Pool ID
4. tag: TAG ID (for sample recognition)
5. sample: Sample ID
6. vector: Vector ID
7. tissue: Biological tissue of the harvest
8. treatment: time point
9. enzyme: Enzyme used (for Linker-Mediated-PCR is NONE)
10. complete_name: Complete name of the sample (link to the association file)
11. header (Primary): Header of the read (from FASTQ)
12. chr: Chromosome
13. integration_locus: Locus of the integration site
14. sequence_count: Count of how many reads are collapsing in the same locus
15. score: Mapping quality
16. strand: Strand of the read ('+' for 5'-3', '-' for 3'-5')
17. label: String
18. sequence_raw: Raw sequence
19. sequence_trimmed: Trimmed sequence

## Integration Site _refactored Table

1. prod_header (Primary): Header of the read (from FASTQ)
2. prod_chr*: Chromosome
3. prod_locus*: Starting locus of the integration site
4. prod_end*: Ending locus of the integration site
5. prod_strand*: Strand of the read ('+' for 5'-3', '-' for 3'-5')
6. ref_associationid: Complete name of the sample (link to the association file)
7. ref_matrixid: String
8. ref_poolid: Pool ID
9. isread_chr: Chromosome
10. isread_start: Starting locus of the integration site
11. isread_end: Ending locus of the integration site
12. isread_strand: Strand of the read ('+' for 5'-3', '-' for 3'-5')
13. isread_RG: Read group
14. isread_quality: Mapping quality
15. isread_NM: Number of mismatches
16. isread_flag: BAM alignment flag
17. isread_cigar: CIGAR string
18. isread_MD: MD score from string
19. isread_insert_size: Insert Size
20. isread_AS: Alignment score from BAM
21. isread_XS: Suboptimal alignment score
22. isread_SA: Secondary alignment
23. isread_nasequence: Genomic part of the read (trimmed)
24. mate_chr: Pair chromosome
25. mate_start: Pair starting locus of the integration site
26. mate_end: Pair ending locus of the integration site
27. mate_strand: Pair strand of the read ('+' for 5'-3', '-' for 3'-5')
28. mate_RG: Pair read group
29. mate_quality: Pair mapping quality
30. mate_NM: Pair number of mismatches
31. mate_flag: Pair BAM alignment flag
32. mate_cigar: Pair CIGAR string
33. mate_MD: Pair MD score from string
34. mate_insert_size: Pair insert Size
35. mate_AS: Pair alignment score from BAM
36. mate_XS: Pair suboptimal alignment score
37. mate_SA: Pair secondary alignment
38. mate_nasequence: Pair genomic part of the read (trimmed)

* If the mate (pair) is present these fields are considered for the pair and not for only R1.

## Stats Summary

Moreover, a table containing all the stats of the run is created in the same database.

1. RUN_ID: RUN (VISPA2) ID
2. RUN_NAME: Concatenation of \$DISEASE\$PATIENT\$POOL variables
3. DISEASE: Disease ID
4. PATIENT: Patient ID
5. POOL: Pool ID
6. TAG: TAG ID (for sample recognition)
7. LTR_ID: LTR ID
8. LC_ID: LC ID
9. PHIX_MAPPING: Number of reads mapping on PhiX genome (overall)
10. PLASMID_MAPPED_BYPOOL: Number of reads mapping on plasmid genome by pool
11. RAW_NO_PLASMID: Number of reads not mapping on plasmid genome by pool
12. BARCODE_MUX: Number of reads Demultiplexed reads by sample
13. LTR_IDENTIFIED: Number of reads with LTR identified
14. TRIMMING_LTRR1: Number of reads with LTR identified on R1
15. TRIMMING_LTRR1R2: Number of reads with LTR identified on R1 and R2
16. TRIMMING_LTRR1R2_LCR1: Number of reads with LTR identified and LC on R1
17. TRIMMING_FINAL_RESCUED: Number of reads rescued
18. TRIMMING_FINAL_LTRLC: Number of trimmed reads overall
19. LV_MAPPED: Number of reads mapping LV genome (internal control band)
20. BWA_INPUT: Number of reads in input on BWA-MEM
21. BWA_MAPPED: Number of reads mapped with BWA-MEM
22. BWA_MAPPED_PP: Number of reads mapped with BWA-MEM properly pair
23. BWA_MAPPED_ST: Number of reads mapped with BWA-MEM as singletons
24. BWA_MAPPED_OVERALL: Number of reads mapped with BWA-MEM overall
25. BWA_ALIGNED_R1: Number of reads mapped with BWA-MEM with only R1
26. RECALIB_MAPPED: NONE
27. RECALIB_MAPPED_PP: NONE
28. RECALIB_MAPPED_ST: NONE
29. RECALIB_MAPPED_OVERALL: NONE
30. RECALIB_ALIGNED_R1: NONE
31. RECALIB_SOFCLIPPED_READS: NONE
32. FILTER_MATE_TO_REMOVE: Number of reads filtered by mate program
33. FILTER_CIGARMD_TO_REMOVE: Number of reads filtered by CIGAR program
34. FILTER_JOINT_MC_TO_REMOVE: Number of joint reads filtered by MD
35. FILTER_JOINT_MC_PP: Number of joint properly pair reads filtered
36. FILTER_JOINT_MC_ST: Number of joint singleton reads filtered
37. FILTER_JOINT_MC_OVERALL: Number of joint reads filtered
38. FILTER_JOINT_ALIGNED_R1: Number of joint reads filtered by R1
39. FILTER_ALMQUAL_PP: Number of properly pair reads filtered by alignment quality
40. FILTER_ALMQUAL_ST: Number of singleton reads filtered by alignment quality
41. FILTER_ALMQUAL_OVERALL: Number of reads filtered by alignment quality overall
42. FILTER_ALMQUAL_ALIGNED_R1: Number of reads filtered by alignment quality on R1
43. ISS_FINAL: Number of IS reads (non unique, redundant)
44. ISS_MAPPED: Number of IS reads mapped (non unique, redundant)
45. ISS_MAPPED_PP: Number of properly pair IS reads (non unique, redundant)
46. ISS_MAPPED_ST: Number of singleton IS reads mapped (non unique, redundant)
47. ISS_MAPPED_OVERALL: Number of IS reads mapped (non unique, redundant) overall
48. ISS_ALIGNED_R1: Number of IS reads mapped (non unique, redundant) on R1

# Heuristic Integration Site Merging

We developed a tool in Python downloadable here (mercurial repository): <https://bitbucket.org/tigetbioinformatics/integration_analysis>

**create_matrix** --dbDataset “patients.mld” --columns sample,tissue,treatment,vector,enzyme --IS_method classic --bp_rule 7 --collision --tsv --no_xlsx

1. *-dbDataset*: Datasets to analyze (tables must be present on MySQL Database) and to collide.
2. *-columns*: Indicate the columns for the final matrix output. Available fields (from the association file): n_LAM, tag, pool, tissue, sample, treatment, group_name and enzyme.
3. *-IS_method*: Specify which method run to retrieve Integration Sites: 'classic' or 'gauss' (strand_specific only). You'll be able to tune 'classic' through --bp_rule (default provided); 'gauss' method has to be set-up through --interaction_limit and --alpha (no defaults provided for it).
   1. *Classic*: All reads that are in the same window are merged into a single locus, represented by the mode in the window.
   2. *Gauss*: *interaction limit* states, *de facto*, the number of bin of the histogram you get. Alpha states how many Δ are equal to half-base pair. Some examples: alpha = 1 means that Δ is half-bp long; then 3bp are long 6Δ, α = 0.5 means that Δ is 1-bp long.
4. *-bp_rule*: Minimum number of empty base-pairs between reads belonging to different cluster (also called Covered Bases Ensembles). If you chose 'classic' method to retrieve IS, this number also set the maximum dimension allowed for a *Covered Bases/Ensemble* (n+1 bases). Default option is '3', i.e. 'minimum 3 empty-bp between independent ensembles, an ensemble can span at most 4bp'. Conversely, if you chose 'gauss' method, it will be automatically set equal to *interaction limit* (overriding your setting) and no limit of dimension will be set for ensembles construction.
5. *-collision*: Produces “collisions” between one dataset and a list of some others. All datasets versus each other. For each dataset, over current, is hung at the end of a column matrix containing for each integration, how many have been found in comparison datasets. Each IS is compared between datasets with a radius equal to *bp_rule*+1.
6. *-tsv*: Produces output matrixes in tab-separated format (UTF-8 encoded).
7. *-no_xlsx*: With this option no excel files are produced.

The program has also some default parameters like *host* (IP address to establish a connection with the server that hosts DB, localhost), *user* (username to log into the server you just chosen through, readonly), *pw* (password for the user you choose to log through, readonlypswd), *dbport* (database port, 3306), *query_steps* (number of row simultaneously retrieved by a single query, 50000000), *reference_genome* (specify reference genome, hg19), *strand_aspecific* (if called, strands will be merged together instead of be treated separately), *set_radius* (along with --collision option, here you can set the maximum distance (i.e. loci difference) between two covered bases regarded as ‘colliding’, None), *diagnostic* (XLSX output will be created without any frills but equipped with specific formulas to perform output control, self-coherence and DB coherence), *statistics* (statistical report will be created, equipped with graphs and many more features constantly developing (bioinfo-oriented). By default, this report is an Excel Workbook file (*.xlsx) but a *.tsv version (less featured) is also available, using --tsv option).

An example of a matrix file is reported in **Figure S6**.


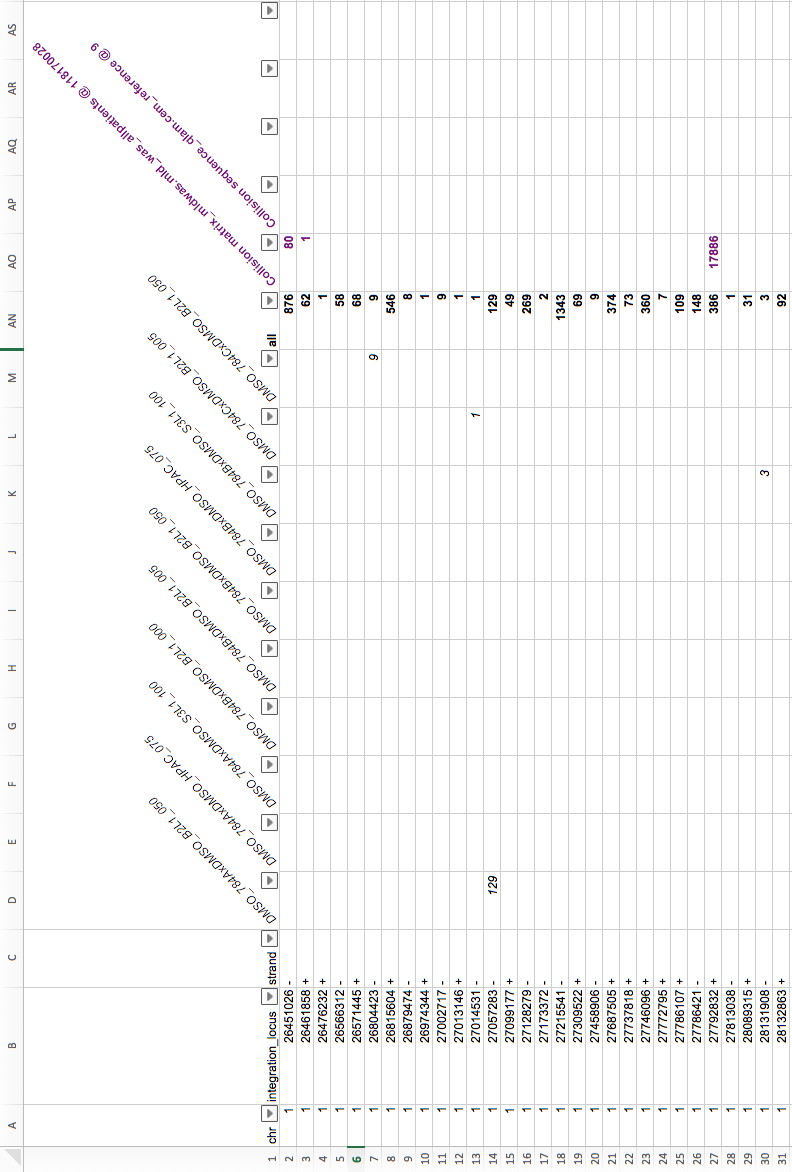


**Figure S6:** IS Matrix, a sample picture extracted from the output of VISPA2 for anti-cancer drug resistance, example taken from [8]. Each row contains an IS (with genomic coordinates, columns A-C) whereas columns (from D to M) represent the processed samples. If an IS has been retrieved for a given sample, then the cell contains the number of reads mapping in that IS (referred as sequence count).

# Integration Site Annotation

To each identified IS can be associated to nearby genomic annotations such as genes, miRNAs and potentially other annotations. For this task, we developed an annotation tool (**annotate_matrix):**

**annotate_matrix** [-m $IS_MATRIX.TSV] [-t $TYPE] [-g $GTF_FILE.GTF] [-o $OUTPUT_DIR]

1. *-m* [IS_MATRIX.TSV]: IS matrix file (tab-separated).
2. *-t* [TYPE]: VISPA2 Matrix file.
3. *-g* [GTF_FILE.GTF]: Positions of all data items in a standard gene prediction format (similar to a BED file, downloadable from: <https://genome.ucsc.edu/cgi-bin/hgTables> but with added lines with chrM for annotation).
4. *-o* [OUTPUT_DIR]: Output directory

# References

1. Brown J, Pirrung M, McCue LA: **FQC Dashboard: integrates FastQC results into a web-based, interactive, and extensible FASTQ quality control tool**. *Bioinformatics* 2017.

2. Firouzi S, Lopez Y, Suzuki Y, Nakai K, Sugano S, Yamochi T, Watanabe T: **Development and validation of a new high-throughput method to investigate the clonality of HTLV-1-infected cells based on provirus integration sites**. *Genome Med* 2014, **6**(6):46.

3. Bolger AM, Lohse M, Usadel B: **Trimmomatic: a flexible trimmer for Illumina sequence data**. *Bioinformatics* 2014, **30**(15):2114-2120.

4. Calabria A, Spinozzi G, Benedicenti F, Tenderini E, Montini E: **adLIMS: a customized open source software that allows bridging clinical and basic molecular research studies**. *BMC Bioinformatics* 2015, **16 Suppl 9**:S5.

5. Dodt M, Roehr JT, Ahmed R, Dieterich C: **FLEXBAR-Flexible Barcode and Adapter Processing for Next-Generation Sequencing Platforms**. *Biology (Basel)* 2012, **1**(3):895-905.

6. Li H, Durbin R: **Fast and accurate short read alignment with Burrows-Wheeler transform**. *Bioinformatics* 2009, **25**(14):1754-1760.

7. Li H, Handsaker B, Wysoker A, Fennell T, Ruan J, Homer N, Marth G, Abecasis G, Durbin R, Genome Project Data Processing S: **The Sequence Alignment/Map format and SAMtools**. *Bioinformatics* 2009, **25**(16):2078-2079.

8. Ranzani M, Annunziato S, Calabria A, Brasca S, Benedicenti F, Gallina P, Naldini L, Montini E: **Lentiviral Vector-based Insertional Mutagenesis Identifies Genes Involved in the Resistance to Targeted Anticancer Therapies**. *Molecular Therapy* 2014.
